# Reliability Evaluation and Robust Design of a Sensor in an Entire Roller-Embedded Shapemeter

**DOI:** 10.3390/s18071988

**Published:** 2018-06-21

**Authors:** Haimiao Wu, Guohua Cui, Dan Zhang, Hongmin Liu

**Affiliations:** 1 College of Mechanical and Equipment Engineering, Hebei University of Engineering, Handan 056038, China; wuhaimiao@163.com; 2 Department of Mechanical Engineering, Shanghai University of Engineering Science, 333 Longteng Road, Shanghai 201620, China; 3 National Engineering Research Center for Equipment and Technology of Cold Rolling Strip, Yanshan University, Qinhuangdao 066004, China; Liuhm@ysu.edu.cn

**Keywords:** shapemeter roll, sensor, reliability evaluation, reliability robust design, initial interference

## Abstract

The intermittence of the shape detection signal associated with an entire roller-embedded shapemeter roll, used in a seven-pass cold reversible rolling process, is considered. A transient interference at the sensor top surface and the distance between the sensor top surface and the roll outer surface are developed, and a sensor reliability evaluation model is derived. The reliability of the sensor is evaluated via the random perturbation method, and the reliability sensitivity of design variables is proposed. The analysis reveals that the reliability is smallest in the third rolling pass. Of the design variables considered, the initial interference exhibits the largest reliability sensitivity and has the greatest influence on the sensor reliability. A reliability robust design model of the initial interference is therefore developed. A new shapemeter roll is fabricated and tested in a 1050 reversible cold rolling mill. The test results are consistent with the theoretical results, thereby validating the proposed model. The selection of an appropriate initial interference provides an important means of overcoming the adverse effects associated with the thermal deformation of sensor contact surfaces.

## 1. Introduction

With the rapid development of modern cold-rolling technology and the continuous increase in the number of cold-rolled-product specifications, ultra-thin cold-rolled strips with a large width-to-thickness ratio have attracted significant attention. This interest stems from the good economic performance of these strips, the development of shape detection technology and the need for equipment capable of processing ultra-thin cold-rolled strips with large width-to-thickness ratios. Compared with the large-scale cold strip mill, a single-stand reversible cold rolling mill is more suitable for obtaining such strips. These strips must satisfy strict requirements regarding the strip shape index. Development of a shapemeter, which is suitable for the single-stand reversible cold rolling mill, is essential for developing these strips [1]. The shapemeter roll is the main component of the shapemeter and plays the key role in on-line shape detection. A rolling cycle of a single-stand reversible cold rolling mill is characterized by temperature variations (by up to 160 °C) of the strip in contact with the roll. This results in a relatively large temperature difference (up to 70 °C) between the temperatures of the strip before and after each rolling pass. The ever-changing strip tension and temperature associated with each rolling pass change the pre-pressure of the sensor in the shapemeter roll. This affects the reliability of the sensor, resulting in an unstable or intermittent shape detection signal [2,3]. Studies focused on the reliability of the sensor have therefore become crucial for improving rolling operations.

The influence of sensor reliability on the shape detection signal has rarely been investigated. Yang et al. [4,5,6] determined the influence of zero residual voltage and the shapemeter roll deflection on the sensor work reliability. However, the influence of the sensor reliability, resulting from the sensor size parameters induced by the temperature and tension of the strip, remains unexplored. Wu et al. [2,3,7,8,9] analyzed the work condition of a shapemeter employed in a seven-pass cold reversible rolling process. In [7], a coupled thermo-mechanical model was developed for analyzing the transient temperature field and the maximum equivalent stress of a shapemeter roll using the finite element commercial software ANSYS (ANSYS, PERA Global Inc., Beijing, China). The analysis revealed that the maximum equivalent stress was considerably lower than the strength required of the material. The work pressure model and pre-pressure model of the shapemeter roll were deduced in [2,3,8,9]. The influence of the strip temperature on the contact status of the interference fit surface was analyzed via the finite element method, and a reasonable range of the interference fit value was determined. These studies focused on the shapemeter roll, but the transient reliability of the sensor has yet to be evaluated, and optimization of the initial interference via the robust design method remains unrealized. Several studies have considered reliability. For example, Zhang et al. [10,11,12] used the random perturbation method to analyze the reliability sensitivity of a mechanical structure system and a series-parallel system and proposed a reliability and robust design. Du et al. [13,14,15] performed time-dependent reliability analysis of mechanical structures by considering the randomness of variables. However, the analysis focused on the reliability of a single physical field, rather than on the thermal-mechanical coupling field problem.

The present work considers the disappearance of the shape signal resulting from the continual change in tension and temperature of a strip in contact with a shapemeter roll. The strip is obtained through a seven-pass cold reversible rolling process. The size parameter model of the sensor in the entire roller-embedded shapemeter roll is developed, and the transient reliability evaluation model of the sensor is derived. Considering the randomness of design variables, the reliability index and reliability of the sensor are determined via the random perturbation method. The reliability sensitivity of the design variables is also obtained. Moreover, a reliability robust design model of the initial interference is built, based on the constraint conditions.

## 2. Application Background

### 2.1. Composition of the Entire Roller-Embedded Shapemeter

**Figure 1:**
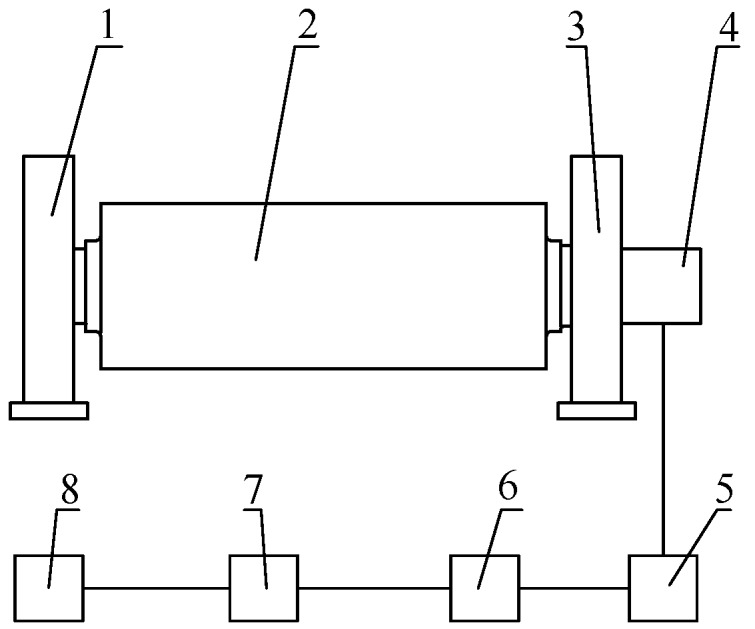
System of entire roller-embedded shapemeter. 1, transmission side bearing seat; 2, shapemeter roll; 3, operating side bearing seat; 4, self-generating signal processing and wireless transmitting device; 5, wireless receiver; 6, decoder; 7, software processing system; 8, memory and peripherals.

As shown in Figure 1, the entire roller-embedded shapemeter includes a transmission side bearing seat, a shapemeter roll, an operating side bearing seat, a self-generating signal processing and wireless transmitting device, a wireless receiver, a decoder, a software processing system, a memory and peripherals. There is no gap on the outer surface of the roll, and the roll consists of two special holes that lie along the axial direction of the roll body. The precision special hole is a through hole, and the sensor is inserted into the precision-deep hole. The sensor signal line passes through this hole, leads to the axle head on the operating side and is connected to the self-generating signal processing and wireless transmitting device. The shape signal detected by the sensor is sent out as a wireless signal through self-generating signal processing and the wireless transmission device. The wireless signal is sent to the receiver, and the decoder restores this signal. The restored signal then enters the software processing system for digital filtering and signal compensation, and the online shape signal is thereby obtained.

**Figure 2:**
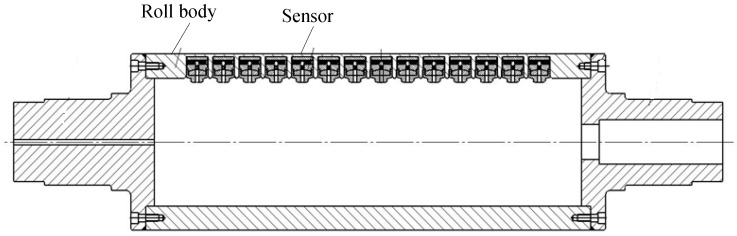
Structure of the entire roller-embedded shapemeter roll.

**Figure 3:**
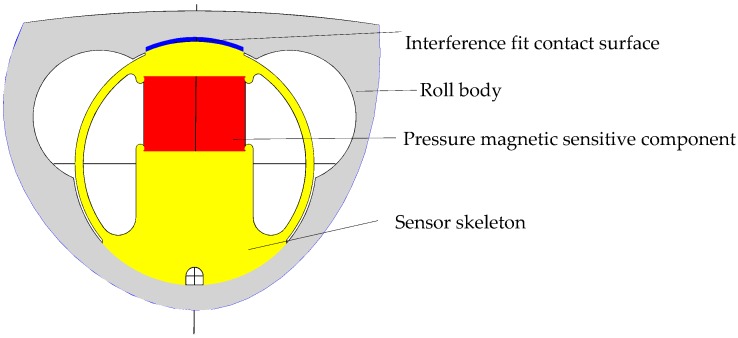
Structure of the sensor in the entire roller-embedded shapemeter roll.

As shown in Figure 2, the entire roller-embedded shapemeter roll (outer diameter: 400 mm, inner diameter: 260 mm) consists of a roll body and sensors. Each sensor (diameter: 40 mm) is composed of a skeleton and a pressure magnetic sensitive component (see Figure 3). The interference between the 5 mm-thick roll body on the top of the sensor and the top surface (sensor contact surface) of the sensor is 0.04 mm. The sensor serves as the key component for measuring the contact pressure of the cold-rolled strip.

### 2.2. Composition of the Pressure Magnetic Sensitive Component

**Figure 4:**
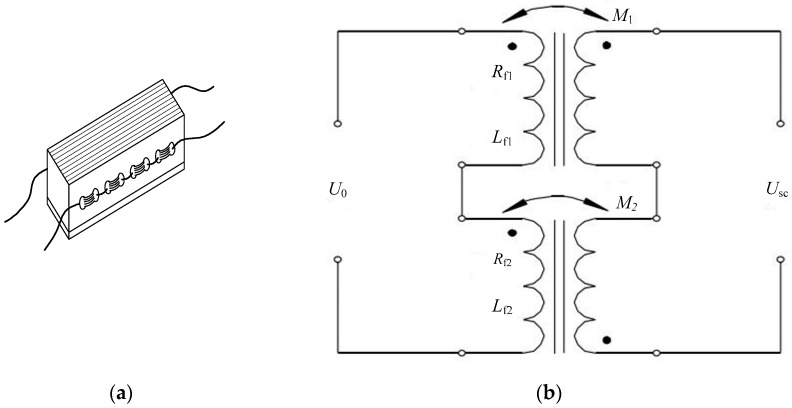
Pressure magnetic sensitive component: (**a**) structure; (**b**) equivalent circuit.

As shown in Figure 4, the primary windings in the two sensors are connected in series, and the secondary windings are connected in opposite phases, thereby forming a differential circuit. Compared with other connection methods, this connection method can yield a higher sensitivity output signal, eliminate the influence of sensor pre-pressure on the sensor output signal, reduce the influence of the temperature and centrifugal force of the roll on the sensor output signal and solve the problem of sensor signal zero drift. Therefore, the shapemeter roll is well suited to the harsh work environment and can improve the accuracy of the detection signal, thereby ensuring the reliable and stable long-term operation of the roll.

### 2.3. Strip Shape Detection Principle

**Figure 5:**
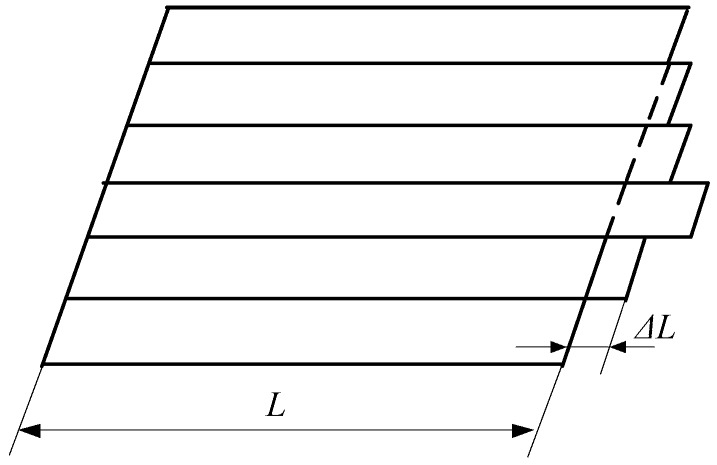
Extension of the strip.

As shown in Figure 5, the strip is divided into several longitudinal narrow strips along the width direction, and each narrow strip is interacted with and interacts with each other. Unequal elongation of these strips leads to interaction among the strips, and a stress, i.e., a residual stress, is thereby generated. Tensile stress components and compressive stress components of this stress occur along the transverse direction of the strip. When the compressive stress is greater than the critical buckling stress, the strip undergoes buckling, undulation and arching. If the compressive stress is smaller than the critical buckling stress, the strip remains flat in appearance, but the internal residual stress of the strip still produces a potential shape. The nature of the shape corresponds to the distribution of the residual stress along the width of the strip, and hence, the residual stress can be used to represent the shape of the strip. The transverse distribution of the residual stress of the strip is given as follows:

(1) $$\Delta \sigma \left(y\right)=\sigma \left(y\right)-\bar{\sigma }$$

where Δσ(y), σ(y) and $\bar{\sigma }$ are the residual stress, front tension stress and average front tension stress, respectively.

**Figure 6:**
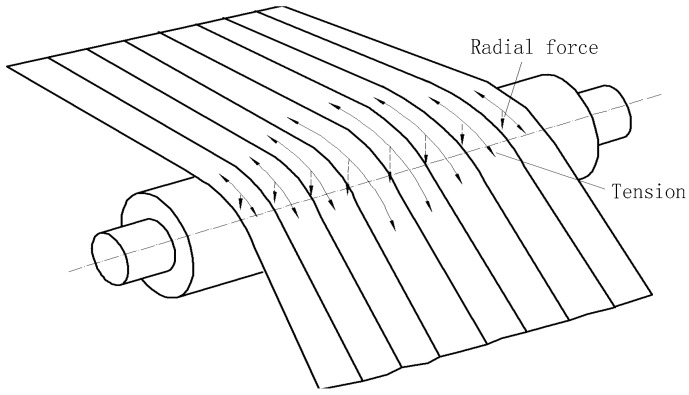
Detection principle of the entire roller-embedded shapemeter.

As shown in Figure 6, on-line measurements of the non-uniform longitudinal extension of the strip are impossible, but the strip shape can be described by the distribution of the transverse tensile stress in the strip. During the rolling process, the strip is tight on the shapemeter roll. The longitudinal tensile stress and residual stress of the strip are therefore converted into the pressure of the shapemeter roll outer surface. The on-line shape of the strip can then be determined by measuring this pressure [1,2]. The entire roller-embedded shapemeter plays a critical role in measuring the on-line pressure of the strip.

**Figure 7:**
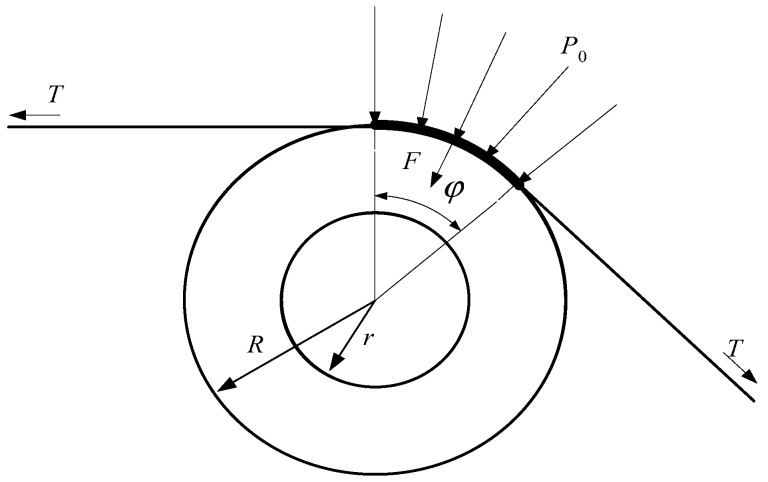
Forces acting on the roll of the entire roller-embedded shapemeter roll.

As shown in Figure 7, the residual stress of *i*-th sensor is:

(2) $$\Delta \sigma_{i}=\frac{F_{i}-\bar{F}}{\bar{F}}\frac{T}{Bh}$$

where, T, $F_{i}$, B, $\bar{F}$ and h are the total strip tension, residual pressure detected by *i*-th sensor, strip width, average value of the radial pressure detected by the sensors and strip thickness, respectively.

### 2.4. Problem Introduction and Research Significance

The temperature at each rolling pass of the strip in contact with the shapemeter roll changes frequently, and the temperature field of the roll changes constantly. Therefore, the contact state of the interference fit surface at the sensor top surface also changes constantly, leading to variations in the sensor pre-pressure at the top of the sensor. If the pre-pressure decreases to zero, the sensor is unable to measure the pressure of the shapemeter roll outer surface, and the shape signal will disappear. Hence, establishing a sensor reliability evaluation model, analyzing the sensitivity of sensor size parameters and designing a sensor via the robust design method are necessary [16,17].

## 3. Sensor Size Parameters Design Model

Sensor size parameters, including the diameter of the sensor, interference at the sensor top surface and distance between the sensor top surface and the roll outer surface, have a significant influence on the sensor reliability. The diameter of the sensor is determined in accordance with the structural design requirements. Therefore, the interference at the sensor top surface and the distance between the sensor top surface and the roll outer surface can be analyzed.

### 3.1. The Transient of Interference at the Sensor Top Surface

According to [8], the pressure applied by the strip to the sensor is governed by:

(3) $$P_{1}=\frac{T}{n}\frac{\frac{e^{4}}{R}}{c^{3}+2e\left(e^{2}-c^{2}\right)+\frac{24E_{2}f^{3}a}{\left(1-\mu_{2}\right)E_{1}c}}$$

where *e* is the width of special hole on the sensor top surface, *R* is the radius of the shapemeter roll, *c* is the contact width between the sensor top surface and the roll body, *b* is the axial length of the sensor, *f* is the distance between the sensor top surface and the roll outer surface, *E*_1_ is the sensor elastic modulus, *E*_2_ is the shapemeter roll elastic modulus, *n* is the number of sensors in the axial direction of the roll body, *µ*_2_ is Poisson’s ratio of the roll body and *a* is the sensor diameter.

Under the total tension of the strip, the deflection of the roll body is governed by:

(4) $$w=\frac{Te^{4}\left(1-\mu_{2}\right)}{32E_{2}f^{3}nbR}$$

The temperature of the contact surfaces at the top of the sensor is determined in accordance with [8,9]. Accordingly, the thermal deformation difference of these surfaces is described by:

(5) $$\delta^{j}\left(t\right)=\alpha_{2}\left(R-f\right)T_{2}^{j}\left(t\right)-\alpha_{1}a\left(1+\mu_{1}\right)T_{1}^{j}(t)-\alpha_{2}\left(R-f-a\right)T_{1}^{j}(t)$$

where $T_{2}^{j}(t)$ is the temperature of the roll body at the top of the sensor in *j*-th rolling pass, $T_{1}^{j}(t)$ is the temperature of the top surface of the sensor in *j*-th rolling pass, $\alpha_{1}$, $\alpha_{2}$ and $\mu_{1}$ are the thermal expansion coefficient of the sensor, the thermal expansion coefficient of the shapemeter roll and Poisson’s ratio of the sensor, respectively.

Based on the geometrical relationship between the sensor top surface and the roll body, the interference at the top of the sensor is described by:

(6) $$\eta^{j}\left(t\right)=\eta_{0}-\delta^{j}(t)+w$$

where $\eta_{0}$ is the initial interference at the top surface of the sensor.

### 3.2. The Distance between the Sensor Top Surface and the Roll Outer Surface

If the influence of the strip temperature on the interference fit is ignored, the sensor is subjected to a certain contact pre-pressure. This pre-pressure (i.e., the initial pre-pressure of the sensor) results from the interference fit between the contact surfaces at the top of the sensor. According to [8], the initial pre-pressure of the sensor is:

(7) $$P_{2}=\frac{\eta_{0}}{\frac{12\left(1-\mu_{2}\right)}{384E_{2}bf^{3}}\left[c^{3}+2e\left(e^{2}-c^{2}\right)\right]+\frac{a}{E_{1}bc}}$$

The initial pre-pressure of the sensor will change, owing to the influence of the strip temperature on the interference fit. The thermal-mechanical coupling of the sensor pre-pressure is:

(8) $$P_{2}{}^{j}\left(t\right)=\frac{\eta^{j}\left(t\right)}{\frac{12\left(1-\mu_{2}\right)}{384E_{2}bf^{3}}\left[c^{3}+2e\left(e^{2}-c^{2}\right)\right]+\frac{a}{E_{1}bc}}$$

According to Equations (7) and (8), the pre-pressure of the sensor will increase with increasing distance between the sensor top surface and the roll outer surface. The equations also indicate that the distance between the sensor top surface and the roll outer surface will have considerable impact on the sensor pre-pressure.

In the work process of the sensor, the work pressure of the sensor must occur along the linear segment of the characteristic curve, otherwise the detection signal will be distorted. From the above analysis, (i) the work pressure of the sensor increases with increasing distance between the sensor top surface and the outer surface of the roll and (ii) the ease with which the detection signal disappears will increase with decreasing distance between the sensor top surface and the outer surface of the roll. Therefore, the distance should be maximized on the condition that the work pressure of the sensor occurs along the linear segment of the characteristic curve. The distance between the top surface of the sensor and the outer surface of the roll is described by:

(9) $$\left\{ \begin{array}{l} \min f(x)=-x\\ s.t.q_{1}\left(x\right)=P_{1}+P_{2}-\left[F_{1}\right]\leq 0\\ \;q_{2}\left(x\right)=\left[F_{2}\right]-P_{1}-P_{2}\leq 0 \end{array}\right.$$

where $P_{1}$ is the pressure that the strip applies to the sensor, $P_{2}$ is the initial pre-pressure of the sensor and $\left[F_{1}\right]$ and $\left[F_{2}\right]$ are the maximum allowable and the minimum allowable pressure of the sensor, respectively.

## 4. Sensor Reliability Evaluation Model

In [7], the transient stress of the shapemeter roll was analyzed using the finite element commercial software ANSYS (ANSYS, Inc.), and the results showed that the maximum stress was substantially lower than the strength of the material. The shapemeter employed in the present study resists strength failure during field testing. In this paper, the reliability analysis is focused only on identifying the reliability mechanism.

### 4.1. Rolling Parameters

The extreme working conditions associated with cold reversible rolling are investigated. Reliability of the sensor under these conditions ensures reliability under less extreme conditions.

The 0.2 mm-thick strip, which is composed of Q195LD steel, was obtained from 2.5 mm-thick raw material subjected to a seven-pass rolling process. The interval between the passes is short and can therefore be neglected. The rolling parameters and the material properties of the shapemeter roll are shown in Table 1 and Table 2, respectively. Moreover, the details of the actual rolling conditions in the field, temperature of the strip in contact with the shapemeter roll and roll outer surface temperature at each rolling pass are measured by an infrared thermal imager and provided in Table 3.

The temperature field of the shapemeter roll must be determined to calculate the: heat transfer coefficient, heat transfer coefficient of contact surfaces at the top of the sensor (*h*_1_) and equivalent heat transfer coefficient of the shapemeter-roll outer surface (*h*_2_). The value of *h*_1_ can be obtained from [18]. In addition, using the inverse heat transfer method [19,20,21], *h*_2_ (see Table 4) can be determined from the (i) temperature of the shapemeter-roll outer surface at the end of each rolling pass and (ii) the temperature of the strip in contact with the roll.

**Figure 8:**
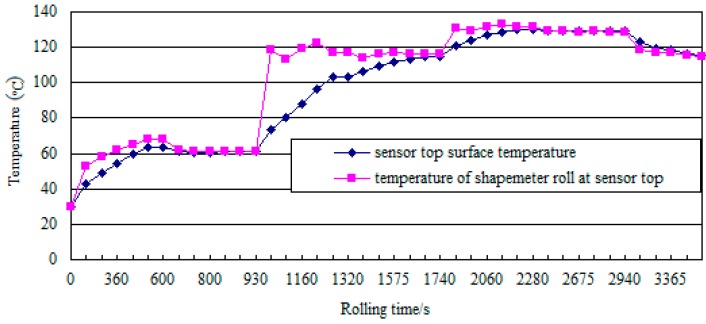
Temperature of the contact surfaces at the top of the sensor.

The temperature of the contact surfaces at the top of the sensor (see Figure 8) is determined via the method described in [9]. As the figure shows, the contact-surface temperature increases initially and then decreases with increasing number of rolling passes. The temperature of the shapemeter roll at the top of the sensor is greater than the temperature of the sensor top surface. This results in a temperature difference between the contact surfaces at the top of the sensor. The maximum difference (~60 °C) occurs in the third rolling pass. Owing to the temperature difference, the thermal-deformation difference between the contact surfaces will increase, whereas the sensor pre-pressure and the reliability of the sensor will decrease.

### 4.2. Limit State Function of Sensor Reliability

If the interference at the top of the sensor is greater than zero during the work process, the pre-pressure of the sensor is greater than zero. If the pre-pressure of the sensor is greater than zero during the work process, the reliability of the sensor will be ensured, and the shapemeter will operate normally. Therefore, the limit state function of the sensor reliability can be derived from Equation (6) and is given as follows:

(10) $$g^{j}\left(X,t\right)=\eta_{0}-\alpha_{2}(R-f)T_{2}^{j}\left(t\right)+\alpha_{1}a\left(1+\mu_{1}\right)T_{1}^{j}(t)+\alpha_{2}(R-f-a)T_{1}^{j}(t)+\frac{Te^{4}\left(1-\mu_{2}\right)}{32E_{2}f^{3}nbR}$$

when $g^{j}\left(X,t\right)>0$, the sensor will operate normally and is non-operational otherwise (i.e., when $g^{j}\left(X,t\right)\leq 0$).

The random variables are considered in accordance with [20], and the reliability of the sensor is evaluated via the random perturbation method. According to [22], the variation coefficient of the geometric dimension and the variation coefficient of the temperature are 0.005 and 0.033, respectively. The respective random variables and the limit state function of reliability are expressed as follows:

(11) $$X=X_{d}+\varepsilon X_{p}$$

(12) $$g^{j}\left(X,t\right)=g_{d}^{j}\left(X,t\right)+\varepsilon g_{p}^{j}\left(X,t\right)$$

where *X_d_*, *X_p_* and *ε* are the definite part of the random variable, the random part of the random variable and a small parameter, respectively.

The first two moments of the limit state function are given as:

(13) $$\mu_{g}=E\left[g^{j}\left(X,t\right)\right]=g_{d}^{j}\left(X,t\right)$$

(14) $$\sigma_{g}^{2}=\mathrm{Var}\left[g^{j}\left(X,t\right)\right]={\left(\frac{\partial g_{d}^{j}\left(X,t\right)}{\partial X^{T}}\right)}^{2}\mathrm{Var}\left(X\right)$$

Similarly, the reliability index *β* is determined from:

(15) $$\beta =\frac{\mu_{g}}{\sigma_{g}}=\frac{E\left(g^{j}\left(X,t\right)\right)}{\sqrt{\mathrm{Var}\left[g^{j}\left(X,t\right)\right]}}$$

The corresponding reliability *Re* is defined as:

(16) Re=Φ(β)

where Φ is the standard normal distribution function.

The reliability sensitivity model that determines the mean and variance of the design variables is governed by:

(17) $$\frac{\partial Re}{\partial X^{T}}=\frac{\partial Re}{\partial \beta }\frac{\partial \beta }{\partial \mu_{g}}\frac{\partial \mu_{g}}{\partial X^{T}}$$

(18) $$\frac{dRe}{d\mathrm{Var}(X^{T})}=\frac{\partial Re}{\partial \beta }\frac{\partial \beta }{\partial \sigma_{g}}\frac{\partial \sigma_{g}}{\partial \mathrm{Var}(X^{T})}$$

where $\frac{\partial \mu_{g}}{\partial X^{T}}=\left[\frac{\partial g}{\partial \eta_{0}}\frac{\partial g}{\partial f}\frac{\partial g}{\partial a}\frac{\partial g}{\partial T_{1}^{j}}\frac{\partial g}{\partial T_{2}^{j}}\right]$, $\frac{\partial \beta }{\partial \sigma_{g}}=-\frac{\mu_{g}}{\sigma_{g}{}^{2}}$, $\frac{\partial \sigma_{g}}{\partial \mathrm{Var}\left(X^{T}\right)}=\frac{1}{2\sigma_{g}}\left(\frac{\partial \mu_{g}}{\partial X^{T}}⊗\frac{\partial \mu_{g}}{\partial X^{T}}\right)$, $\frac{\partial \beta }{\partial \mu_{g}}=\frac{1}{\sigma_{g}}$ and $\frac{\partial Re}{\partial \beta }=\phi \left(\beta \right)$.

### 4.3. Result Analysis and Discussion

**Figure 9:**
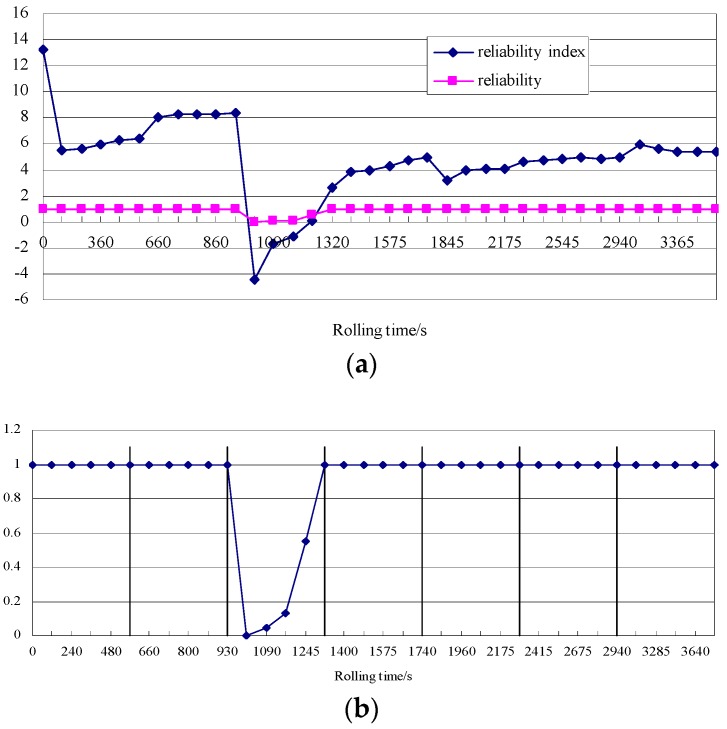
Reliability and reliability index of the sensor: (**a**) reliability index and reliability; (**b**) reliability associated with each rolling pass.

As shown in Figure 9, the sensor reliability and reliability index decreased initially and then increased with increasing rolling time. The reliability and index were lowest in the third rolling pass and close to one for the other rolling passes. The reliability decreased initially and then increased with increasing rolling time in the third rolling pass. At a rolling time of 1005 s, the reliability decreased to the minimum value and was close to zero. At this point, the contact pre-pressure of the sensor was zero; the sensor was non-operational; the shape detection signal disappeared; and the shapemeter malfunctioned. The reliability increased to nearly one at the end of the third rolling pass. At this point, the normal operation of the sensor was restored; the shape detection signal reappeared; and the normal operation of the shapemeter was restored, consistent with the actual operating condition of the shapemeter in the rolling field.

**Figure 10:**
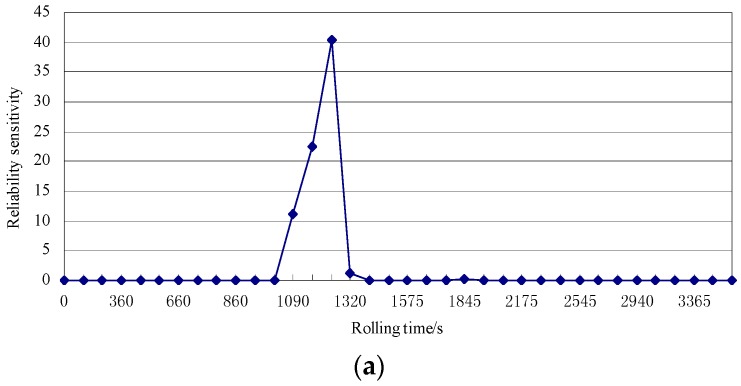

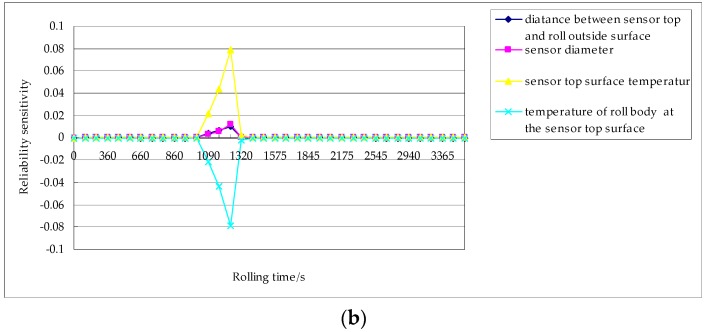
Reliability sensitivity analysis of the mean values of the variables: sensitivity analysis of the (**a**) initial interference; (**b**) other variables.

The reliability sensitivity of the mean values is also considered (see Figure 10). As the figure shows, the reliability of the sensor (i) increased with increasing initial interference, sensor diameter, the distance between the sensor top surface and the outer surface of the roll and the temperature of the sensor top surface and (ii) decreased with increasing temperature of the shapemeter roll at the top of the sensor. The impact of the initial interference on the sensor reliability was greatest in the third rolling pass, where a maximum reliability sensitivity of 40 was realized. Compared with the interference, the other variables have less influence on the reliability of the sensor.

## 5. Reliability Robust Design Model of the Sensor

Analysis of the variable sensitivity revealed that the impact of the initial-interference reliability sensitivity on the sensor reliability was greatest in the third rolling pass. Therefore, the sensor reliability can be improved (through reliability robust design) by adjusting the initial interference.

### 5.1. Mathematical Model of the Reliability Robust Design of the Sensor

The target reliability $Re_{0}$ is 0.999, and the initial interference is designed via the reliability robust design method. The mathematical model for the reliability robust design of the sensor is governed by:

(19) $$\{\begin{array}{c} \min\begin{array}{cc} f(x)=w_{1}f_{1}\left(x\right)+w_{2}f_{2}\left(x\right) & \end{array}\\ \begin{array}{l} s.t.\begin{array}{cc} Re\geq Re_{0} & \end{array}\\ \begin{array}{cc} \begin{array}{cc} & q_{1}\left(x\right)=P_{1}+P_{2}-\left[F_{1}\right]\leq 0 \end{array} & \end{array} \end{array}\\ \begin{array}{ccc} & q_{2}\left(x\right)=\left[F_{2}\right]-P_{1}-P_{2}\leq 0 & \end{array} \end{array}$$

where $f_{1}\left(x\right)$ is the thermal deformation difference between the contact surfaces, $f_{1}\left(x\right)=\alpha_{2}(R-f)\left[T_{2}^{j}\left(t\right)-T_{1}^{j}(t)\right]-a\left[\alpha_{1}\left(1+\mu_{1}\right)-\alpha_{2}\right]T_{1}^{j}(t)$; $f_{2}\left(x\right)$ is the reliability sensitivity of the initial interference, $f_{2}\left(x\right)=\frac{\partial Re}{\partial \eta_{0}}$, w is the weighting factor, $w_{1}=\frac{f_{2}\left(x^{*1}\right)-f_{2}\left(x^{*2}\right)}{\left[f_{1}\left(x^{*2}\right)-f_{1}\left(x^{*2}\right)\right]+\left[f_{2}\left(x^{*1}\right)-f_{2}\left(x^{*2}\right)\right]}$, $w_{2}=1-w_{1}$, and $x^{*1}$ and $x^{*2}$ represent the optimal point of the single objective functions $f_{1}\left(x\right)$ and $f_{2}\left(x\right)$, respectively.

### 5.2. Analysis of the Optimal Result

**Figure 11:**
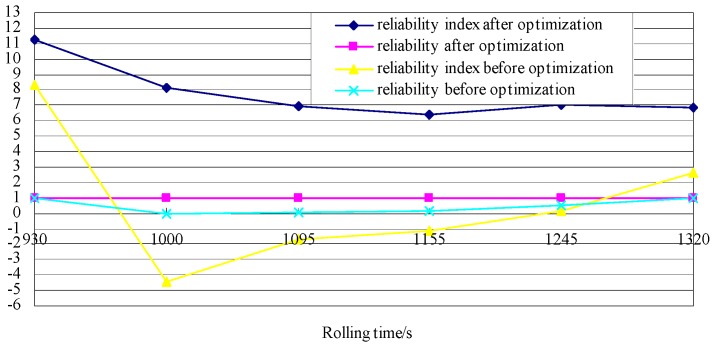
Reliability index, as well as pre- and post-optimization reliability associated with the third rolling pass.

The random perturbation method yields an optimal interference of 0.06 mm. The sensor malfunctions only in the third rolling pass and, hence, only the sensor reliability associated with this pass is evaluated (see Figure 11 for the corresponding results). As the figure shows, the post-optimization reliability index is larger than the pre-optimization value. This post-optimization value is approximately one, thereby satisfying the reliability requirements of the sensor. Therefore, the shapemeter can function normally.

## 6. Test Verification

### 6.1. Manufacture and Installation of the Shapemeter Roll

**Figure 12:**
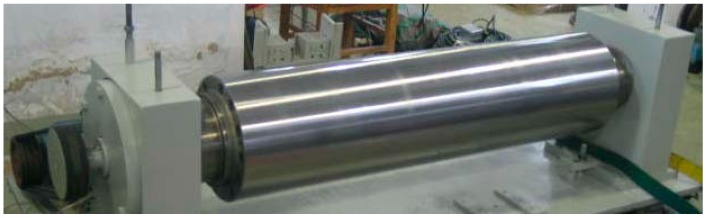
Shapemeter roll prototype.

A new shapemeter roll, with an initial interference value of 0.06 mm, was fabricated as shown in Figure 12. The roll was installed in the position of the guide roll on the outlet side of the reversing cold mill, and the shape signal was detected online while guiding the strip.

### 6.2. Test Process

**Figure 13:**
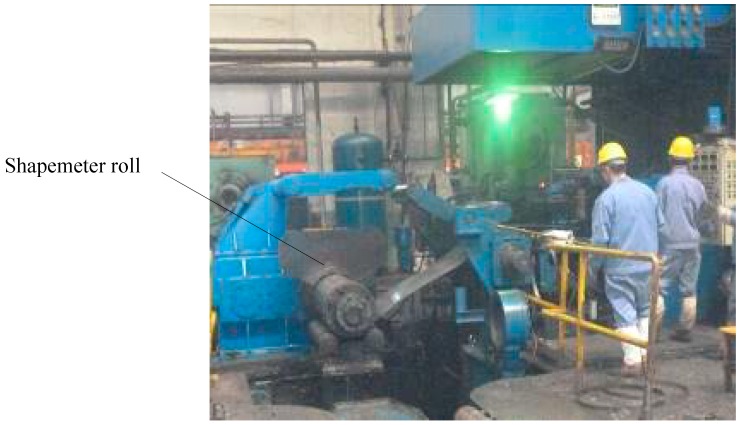
Test field.

**Figure 14:**
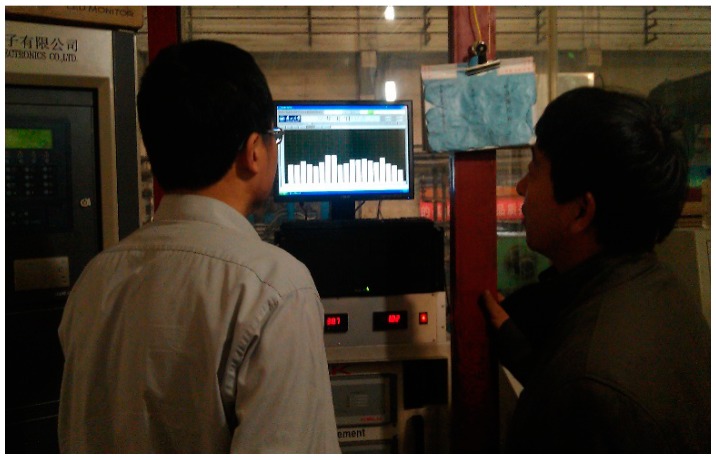
Shape signal processing system.

The 0.2-mm thick strip, which was composed of Q195LD steel, was obtained from 2.5 mm-thick raw material subjected to a seven-pass rolling process. The new shapemeter roll was tested in a 1050 reversible cold rolling mill, as shown in Figure 13. Shape signal processing system and display system are shown in Figure 14.

**Figure 15:**
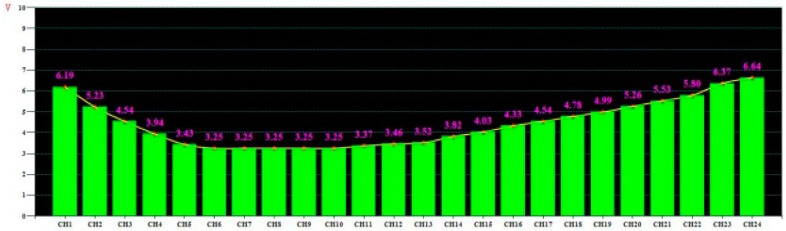
Shape signal detected by sensors embedded in the roll body at a rolling time of 1005 s.

The results of several rolling experiments revealed that the detected signal of the entire rolling process was continuous and complete without any signal discontinuity. The previous analysis revealed that the flatness-detection signal disappeared only in the third rolling pass. Therefore, only a shape detection signal associated with the intermediate rolling time of the third rolling pass (see Figure 15) was obtained.

### 6.3. Test Result Analysis

**Figure 16:**
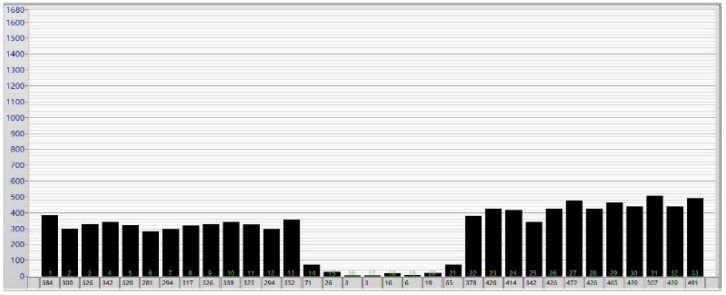
The disappearance of the shape signal.

The disappearance of shape signal is shown in Figure 16. As shown in Figure 15 and Figure 16, the transverse tension of the strip could be detected by the sensor, and the shape detection signal of the third rolling pass was continuous and complete. The signal associated with other rolling passes was also continuous and complete, and hence, the on-line shape of the strip could be exactly determined. Therefore, at an initial interference of 0.06 mm, the problem of intermittent disappearance of the shape detection signal is avoided. Accordingly, reliable operation of the sensor is ensured, and the correctness of the theoretical analysis performed in this work is thereby further validated.

## 7. Conclusions

1. An interference of the sensor top surface is established based on the temperature change and the large tensile force of the strip in the working process. To ensure that the work pressure of the sensor lies within the linear range of the sensor characteristic curve, the distance between the sensor top surface and the roll outer surface is formulated, and the optimum value is obtained.
2. Interference fit surface contact is ensured, by establishing a reliability evaluation model of the sensor. Considering the randomness of design variables, the reliability index and reliability of the sensor are evaluated via the random perturbation method. The reliability sensitivity of the design variables is also determined. The minimum value of the reliability (i.e., approximately zero) occurs in the third rolling pass, and the reliability is almost one in the other rolling passes. Moreover, the maximum reliability sensitivity is realized at the initial interference. Compared with the initial interference, the other design variables exhibit smaller reliability sensitivity, and hence, the (initial) interference is selected for reliability robust optimal design.
3. A reliability robust optimal design model of initial interference is developed, based on constraints that the total pressure and reliability of the sensor must meet the allowable pressure and allowable reliability, respectively. The optimization results reveal a sensor reliability of approximately one for each rolling pass and that the sensor can work normally, at an initial interference of 0.06 mm.
4. A new shapemeter roll, with an initial interference value of 0.06 mm, is fabricated and tested in a 1050 reversible cold rolling mill. The test results revealed that the sensor could work normally and the shape detection signal was continuous and complete in each rolling pass. More importantly, the intermittence of the shape detection signal was eliminated, further validating the correctness of the theoretical analysis performed in this work.

## Figures and Tables

**Table 1:** Rolling parameters.

Rolling Pass	1	2	3	4	5	6	7
**Export strip temperature (°C)**	87	127	130	146	135	151	115
**Reduction ratio (%)**	27.57	29.71	30.89	31.76	31.03	30.00	28.57
**Rolling time (s)**	0–600	600–930	930–1320	1320–1740	1740–2280	2280–2940	2940–3710
**Rolling speed (m·s^−1^)**	3	4.8	7.9	10	10.8	11.2	11.3

**Table 2:** Material properties of the shapemeter roll.

Parameter	Thermal Conductivity (W·m^−1^·K^−1^)	Specific Heat Capacity (J·kg^−1^·K^−1^)	Density (kg·m^−3^)	Coefficient of Thermal Expansion (10^−5^ K^−1^)	Young’s Modulus (10^11^ Pa)	Poisson’s Ratio	Coefficient of Friction
**Roll body**	50	480	7800	1.3	2	0.3	0.2
**Sensor**	50	480	7800	1.3	2	0.3	0.2

**Table 3:** Temperature of the strip in contact with the shapemeter roll and the roll outer-surface temperature at the end of each rolling pass.

Rolling Pass	1	2	3	4	5	6	7
**Temperature of the strip in contact with the shapemeter roll (°C)**	87	62	130	120	135	125	115
**Shapemeter roll outer surface temperature at the end of each rolling pass (°C)**	71	62	122	120	135	130	115

**Table 4:** Equivalent heat transfer coefficient of the roll outer surface.

Rolling Pass	1	2	3	4	5	6	7
**Equivalent heat transfer coefficient of the roll outer surface (10^−6^ W·m^−2^·K^−1^)**	350	350	1667	1500	1700	1500	1400

## Nomenclature

z	The residual stress
σ(y)	Front tension stress
$\bar{\sigma }$	Average front tension stress
T	The total strip tension
$F_{i}$	Residual pressure detected by the sensor
B	Strip width
$\bar{F}$	Average value of the radial pressure detected by the sensors
h	Strip thickness
*e*	Width of special hole on the sensor top surface
*R*	Radius of the shapemeter roll
*c*	Contact width between the sensor top surface and the roll body
*b*	Axial length of the sensor
*f*	Distance between the sensor top surface and the roll outer surface
$E_{1}$	Sensor elastic modulus
$E_{2}$	Shapemeter roll elastic modulus
*n*	Number of sensors in the axial direction of the roll body
$\mu_{2}$	Poisson’s ratio of the roll body
*a*	Sensor diameter
$T_{2}^{j}(t)$	Temperature of the roll body at the top of the sensor in the jth rolling pass
$T_{1}^{j}(t)$	Temperature of the top surface of the sensor in the jth rolling pass
$\alpha_{1}$	Thermal expansion coefficient of the sensor
$\alpha_{2}$	Thermal expansion coefficient of the shapemeter roll
$\mu_{1}$	Poisson's ratio of the sensor
$\eta_{0}$	Initial interference at the top surface of the sensor
$P_{1}$	The pressure that the strip applies to the sensor
$P_{2}$	Initial pre-pressure of the sensor
$\left[F_{1}\right]$	The maximum allowable pressure of the sensor
$\left[F_{2}\right]$	The minimum allowable pressure of the sensor
*X_d_*	The definite part of the random variable
*X_p_*	Random part of the random variable
*ε*	A small parameter
*β*	The reliability index
*Re*	The corresponding reliability
Φ	The standard normal distribution function
$f_{1}\left(x\right)$	Thermal deformation difference between the contact surfaces
$f_{2}\left(x\right)$	Reliability sensitivity of the initial interference
w	Weighting factor
$x^{*1}$	Optimal point of the single objective functions $f_{1}\left(x\right)$
$x^{*2}$	Optimal point of the single objective functions $f_{2}\left(x\right)$
